# Electrospun Poly(lactide-co-glycolide-co-ε-caprolactone) as a Bioresorbable Platform for Docetaxel-Degradation, Drug Delivery Study and Anticancer Activity

**DOI:** 10.3390/ijms27219789

**Published:** 2026-10-31

**Authors:** Joanna Jaworska, Arkadiusz Orchel, Anna Kaps, Marzena Jaworska-Kik, Mateusz Stojko, Monika Musiał-Kulik, Bożena Kaczmarczyk, Małgorzata Pastusiak, Janusz Kasperczyk

**Affiliations:** 1 Centre of Polymer and Carbon Materials, Polish Academy of Sciences, M. Curie-Skłodowskiej 34, 41-819 Zabrze, Poland; mmusial@cmpw-pan.pl (M.M.-K.); bkaczmarczyk@cmpw-pan.pl (B.K.); mpastusiak@cmpw-pan.pl (M.P.); jkasperczyk@cmpw-pan.pl (J.K.); 2 Department of Biopharmacy, Faculty of Pharmaceutical Sciences in Sosnowiec, Medical University of Silesia, Jedności 8, 41-200 Sosnowiec, Poland; akaps@sum.edu.pl (A.K.);; 3 Scientific Research Facility, Branch in Bielsko-Biala, Medical University of Silesia, 40-055 Katowice, Poland; mateusz.stojko@sum.edu.pl

**Keywords:** electrospinning, drug delivery, docetaxel, prostate cancer, electrospun implants

## Abstract

Drug delivery systems are constantly being developed or modified to meet various requirements, but most of all, they are designed to provide controlled, sustained, and targeted drug delivery. A popular technique of the last few years involves the encapsulation of drugs into polymeric nanofibers with the use of electrospinning. The parameters of the process strongly influence not only the morphology of the fibers but the properties of the whole electrospun system, including the drug release profile. In this study, poly(lactide-co-glycolide-co-ε-caprolactone) and docetaxel were used for the electrospinning process in combination with different kinds of solvents to obtain implantable drug delivery systems. We examined the drug release profile, anticancer activity and course of the nonwoven degradation. As a result, a docetaxel-loaded electrospun terpolymer platform was developed as a drug delivery system intended for potential, loco-regional application in prostate cancer therapy. We observed that the drug release profile of the system prepared using hexafluoroisopropanol differed from the system prepared using dichloromethane, although nearly 100% of the docetaxel was released within 20 weeks in both cases. By contrast, the courses of degradation were similar, except during the last period. Drug-loaded electrospun systems exerted a strong cytotoxic effect on DU-145 and PC-3 prostate cancer cell lines. The flexibility of the system, arising from its fibrous structure, and the anticancer activity observed in vitro provide a basis for further in vivo studies, assessing its suitability as an implantable platform for local drug delivery at the site of tumor growth.

## 1. Introduction

Novel drug delivery systems have been designed to provide controlled, sustained, and targeted drug delivery [1], to overcome drawbacks of conventional drug formulations like fluctuating plasma drug concentrations [2]. The popular technique of the last few years involves the encapsulation of drugs into polymeric nanofibers with the use of electrospinning [3,4]. The formation and morphology of the fibers depend on various parameters such as: solution properties (molecular weight and viscosity, surface tension, solution conductivity, dielectric effect of the solvent), process parameters (voltage, feed rate, temperature, effect of the collector, distance between the tip and the collector), and ambient conditions (humidity, temperature, pressure) [5]. These parameters strongly influence not only the morphology of the fibers but the properties of the entire electrospun drug delivery system (DDS), including the release profile.

Various kinds of release profiles from nanofibers have been obtained and described in the literature, including fast, sustained, prolonged, delayed-release, on-demand release, the release of multiple phases, and the co-delivery of multiple components [6]. By implementing one or more controlled-release strategies during electrospinning, the properties of the DDS can be modulated. Although various fiber morphologies can be obtained during electrospinning, smooth and continuous nanofibers are the most prevalent. In some cases, single-fluid electrospinning can create beaded structures. Formation of the bead occurs on the fiber strings. Beaded nanofibers are usually undesirable; however, in some drug delivery strategies beads can act as a drug reservoir. Thus, electrospinning offers multiple possibilities in drug delivery. Electrospinning has also been used for developing drug-releasing biomaterials, including implants [7,8]. Drug-releasing implants provide many advantages, especially in anticancer therapy. Their extraordinary properties are connected with the ability to provide a high local drug concentration with a very low systemic level. It is particularly important when hazardous drugs are used. Many anticancer therapies involve resections of the tumor occurring within, e.g., breast, prostate, or bone. The resection is always connected with the risk of positive margins. In such cases, drug-releasing implants that act locally are advantageous, and might find application in loco-regional therapy.

Taxanes are diterpenoid substances, which are also called microtubule stabilizers since the mechanism of their action is connected with tubulin binding and enhanced microtubule polymerization [9,10]. Among the taxane group we can distinguish paclitaxel, docetaxel and cabazitaxel. They are used in first-line therapy for breast cancer as well as in ovarian, prostate, head and neck, and non-small-cell lung carcinoma [11]. Although they significantly enhance the overall survival rate of cancer patients, there are some limitations in their use: very poor water solubility and the occurrence of severe side effects. Taxanes can be used in a different formulations [12,13], e.g., as liposomes [14] or micelles [15]. These kinds of drugs are currently being evaluated in different clinical trials [16]. The strategy used is mostly based on so-called “combination therapy” involving different active substances or drugs [17,18].

The most popular aliphatic polyester is poly(lactide-co-glycolide) (PLGA). PLGA has been successfully used for drug delivery devices, which is attributed not only to its properties, but also to its commercial availability. However, the synthesis of more advanced polymers, using three different kinds of structural units, can provide interesting polymeric materials with combined features. Copolymerization is a well-known strategy, and is frequently used to obtain components/materials with moderate properties [19,20]. By introducing additional structural units into the polymeric chain, we can modulate the properties of the polymer and influence the course of degradation and drug release. We have used different self-synthesized copolymers and terpolymers in our previous research to obtain drug delivery systems for various applications. We have developed a paclitaxel delivery system based on a blend of copolymers [21], as well as docetaxel and cabazitaxel delivery systems and implants [22,23], with the use of different copolymers and terpolymers.

In our latest published study, we developed different kinds of electrospun DDS, concomitantly with a preliminary investigation of their anticancer potential [22]. In the current work, the scope of research on such biodegradable nonwovens has been substantially expanded to include several of their additional structural and functional features. We decided to use poly(lactide-glycolide-ε-caprolactone) since polymers with three different kinds of structural units offer promising possibilities for modulating drug release. This idea was combined with another strategy for tailoring drug release—we used two different solvents for the electrospinning process. In the present study, a detailed analysis of the drug release, cytotoxic activity, drug/polymer interactions, fiber morphology, and course of the carrier degradation is presented.

To summarize, bioresorbable, flexible and easily implanted drug-eluting biomaterial was obtained. The designed implant is intended for potential use in loco-regional prostate cancer therapy.

## 2. Results

### 2.1. Morphology of Fibers in Drug-Loaded Electrospun Systems

Poly(lactide-co-glycolide-co-ε-caprolactone) and docetaxel were used to prepare the electrospun mats in the form of patches. Two different solvents were used to prepare the solution for electrospinning, namely, hexafluoroisopropanol (HFIP) and dichloromethane (DCM), in order to compare the effect of the solvent on the properties of the obtained drug delivery systems. It is worth mentioning that in order to obtain a good fiber-forming polymer solution for electrospinning, it is important to choose an appropriate concentration. This requires adjusting the process parameters. Since we used two different solvents in our study, different concentrations of the solution were chosen (after optimization) and different electrospinning parameters (presented in Table 2).

**Figure 1:**
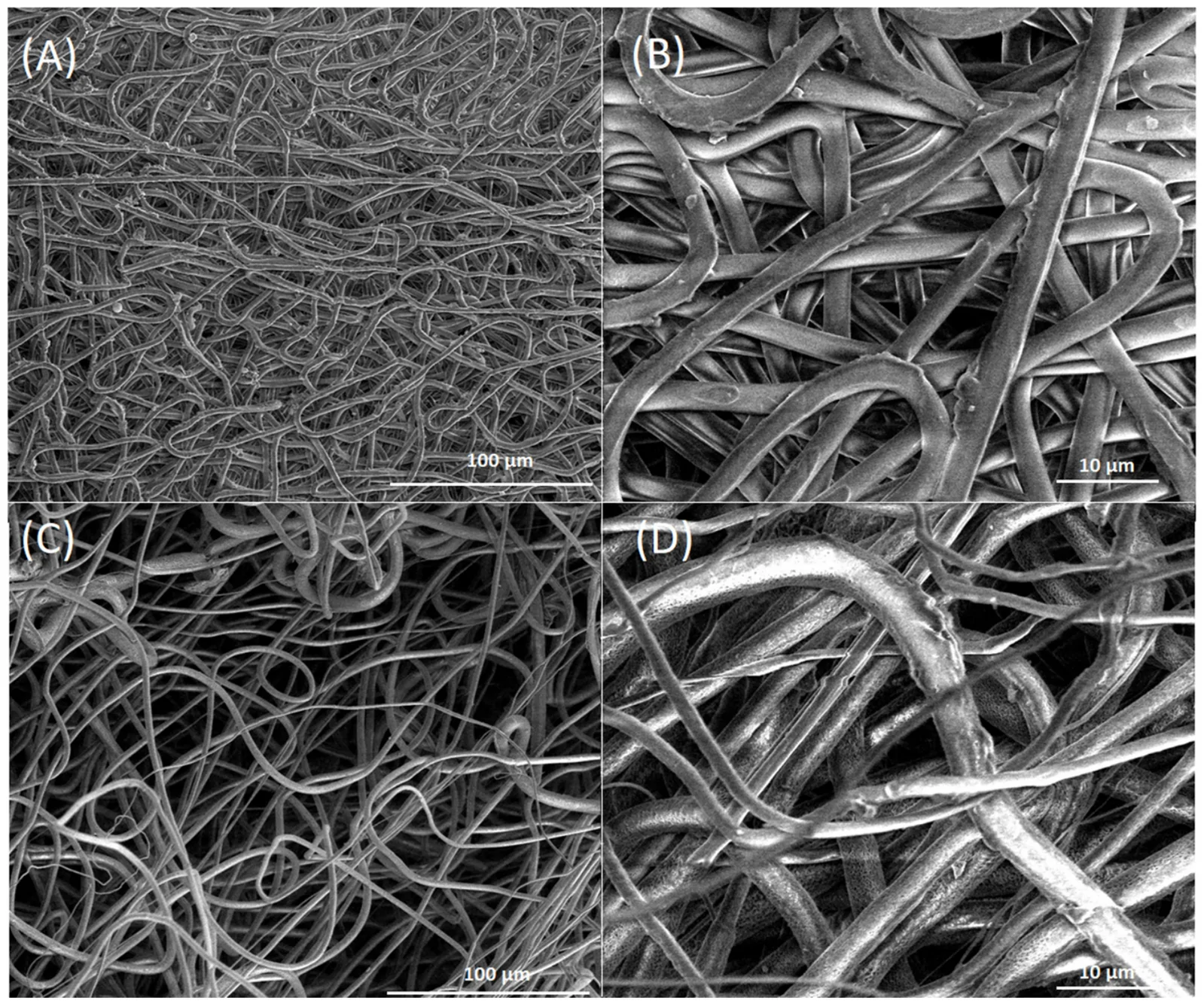
SEM images of drug-loaded patches obtained from poly(lactide-co-glycolide-co-ε-caprolactone) and docetaxel: (**A**,**B**) PLGCL+DTX/HFIP; (**C**,**D**) PLGCL+DTX/DCM.

The final mats are compared and presented in Figure 1. In the case of PLGCL+DTX/DCM, the difference in the diameter of the fibers is noticeable (mean fiber thickness = 3.52 µm, SD = ±1.30 µm). In the case of PLGCL+DTX/HFIP, fibers are more homogeneous (fiber thickness = 2.72 µm, SD = ±0.53 µm).

### 2.2. Interactions Between Polymer and Docetaxel

Interactions between the polymer and docetaxel were evaluated with FTIR spectroscopy. Two regions of the FTIR spectra were analyzed in detail: the region corresponding to the stretching vibrations of the OH and NH groups (3600–3200 cm^−1^), and the region arising from the stretching vibrations of the C=O ester, ketone and urethane groups (1800–1500 cm^−1^).

In the case of pure DTX, a broad band with a maximum at 3453 cm^−1^ and a shoulder at 3340 cm^−1^, corresponding to vibrations of the bonded OH and NH urethane groups, respectively, appeared. At about 3530 cm^−1^, only a low-intensity shoulder was recorded, proving the presence of a small number of free OH groups. Similarly, in the region corresponding to the stretching vibrations of the C=O ester, urethane and ketone groups, a broad band with maxima at 1708 cm^−1^, 1718 and 1737 cm^−1^ was detected. The first two shoulders result from the vibrations of the associated and free C=O urethane groups, respectively, while the third one relates to vibrations of the C=O ester groups. This indicates that in this case, the OH and NH groups formed the hydrogen bonds with C=O in the ester and urethane groups.

**Figure 2:**
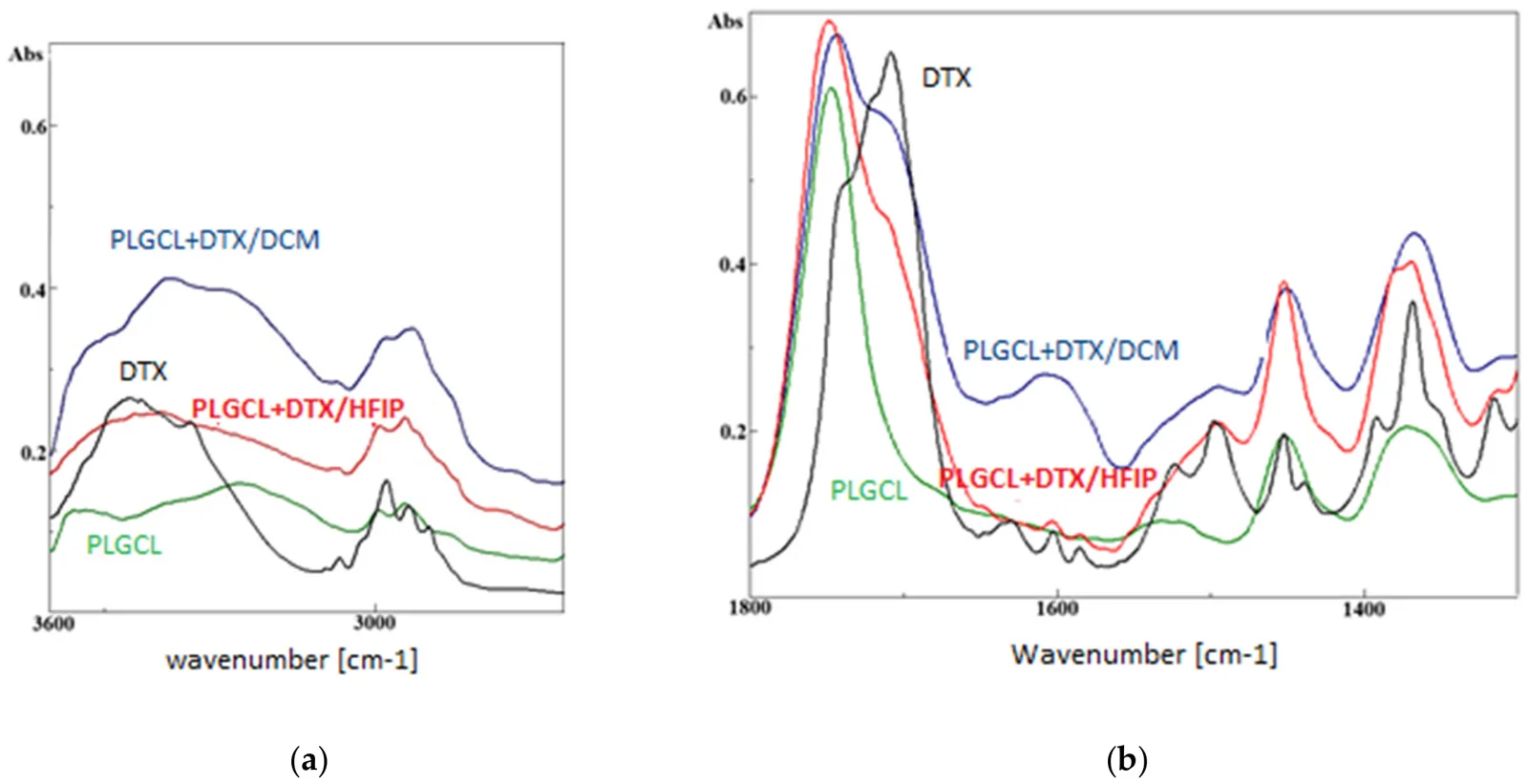
FTIR spectra of drug −free PLGCL, DTX and PLGCL+DTX obtained from DCM and HFIP solvents, (**a**) the range of 3600 ÷ 2500 cm^-1^; (**b**) the range of 1800 ÷ 1300 cm^-1^.

The PLGCL spectrum (Figure 2) showed characteristic bands for polyester, with the most intense band recorded at 1746 cm^−1^ due to the stretching vibrations of the C=O ester groups. The FTIR spectra of PLGCL+DTX/HFIP and PLGCL+DTX/DCM were not the sum of the spectra of particular components. This indicates that some interactions between particular components took place. In both cases, the bands in the region of 3600–3200 cm^−1^ were broadened mainly toward lower wavenumbers, which indicates that more OH and NH groups from DTX formed hydrogen bonds. The observed changes were higher in the case of PLGCL+DTX/DCM. In the region of 1800–1500 cm^−1^ the changes were more difficult to interpret due to the overlapping bands originating from PLGCL+DTX.

The differences observed in the analyzed spectra were caused by the change in the distribution of hydrogen bonds after mixing the PGLCL with DTX.

### 2.3. In Vitro Drug Release Study

Drug loading was evaluated according to three different extraction protocols. (1) Electrospun patches were dissolved in 1 mL of DCM, the mixed for 1 h. After that, 9 mL of ethanol was added and mixed for 1 h. (2) Electrospun patches were dissolved in 10 mL of ACN, then mixed for 1 h. (3) Electrospun patches were dissolved in 5 mL of ACN, then mixed for 1 h. In all cases, the samples were centrifuged at 10,000 rpm for 5 min, and the supernatants were filtered through syringe filters of 0.45 μm and evaluated by means of high-performance liquid chromatography. The efficiency of the procedures used for the extraction of the drug was compared (Table 1).

**Figure 3:**
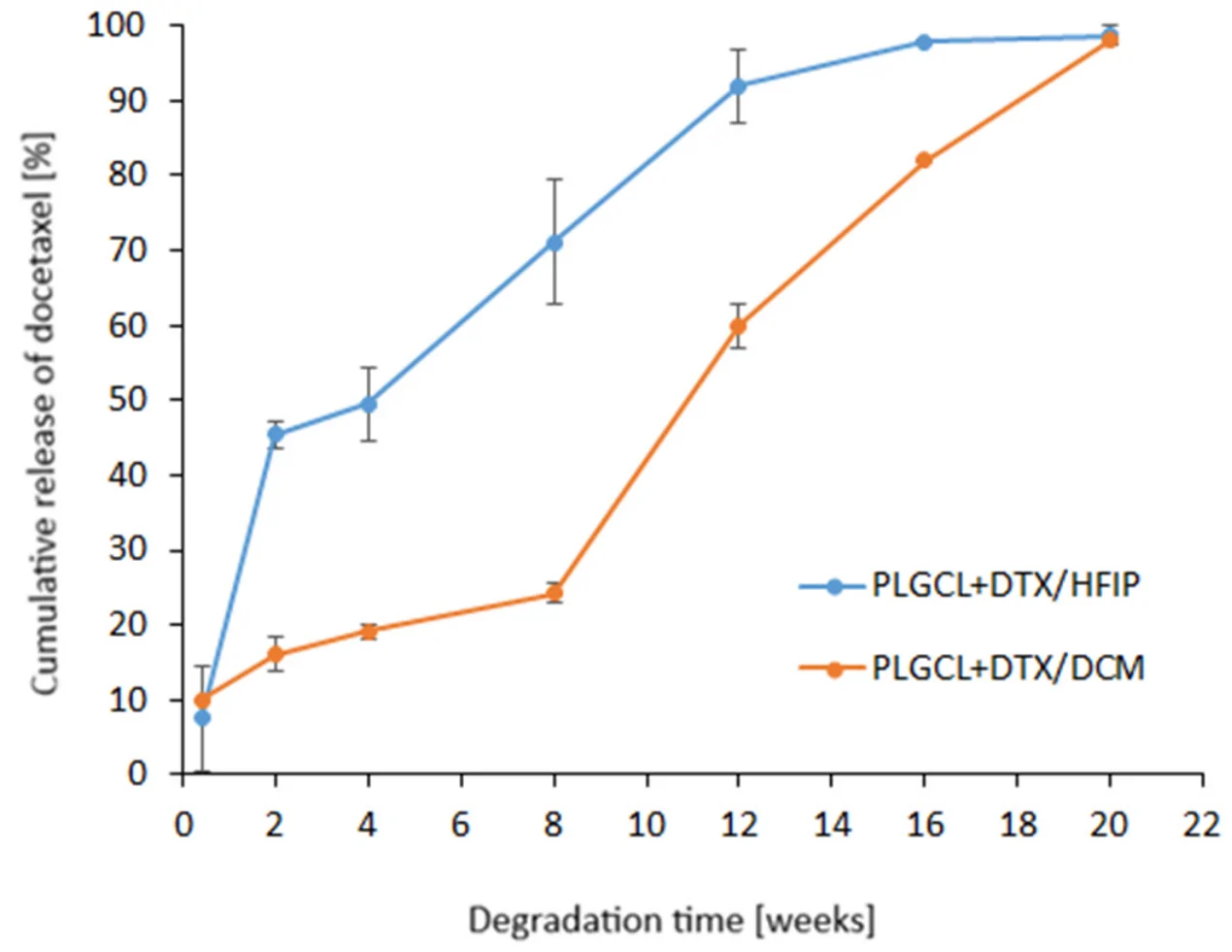
The cumulative release of the docetaxel from poly(lactide-co-glycolide-co-ε-caprolactone) electrospun patches prepared with different solvents (*n* = 3).

Procedure no. 2 was also used to determine the amount of the drug remaining in the electrospun patches after incubation in the degradation medium. Then, this procedure was used to evaluate the cumulative release of the drug. The drug release profiles for PLGCL+DTX/HFIP and PLGCL+DTX/DCM differed. The release of the drug from the PLGCL+DTX/HFIP system was more rapid, especially in the first two weeks of incubation. Within 20 weeks, almost 100% of the docetaxel was released from both electrospun systems (Figure 3).

### 2.4. In Vitro Degradation Process

The course of degradation of the polymer influences the release process of the incorporated drug in polymer-based drug delivery systems. Thus, the detailed characterization of changes during this process needs to be described.

**Figure 4:**
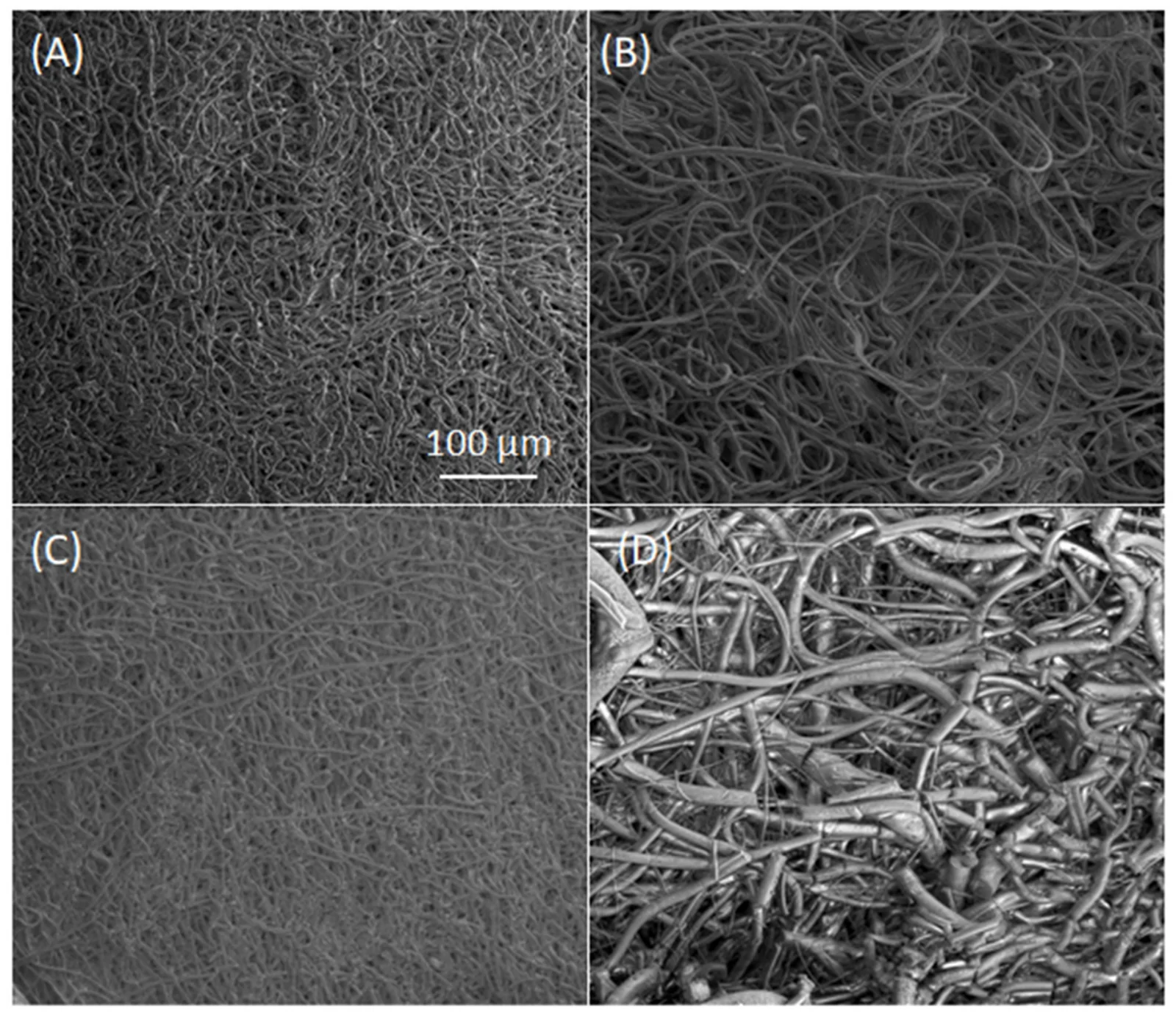
SEM images of drug-loaded patches during degradation (×1000, all scale bars are 100 µm): PLGCL+DTX/HFIP patches: (**A**) 4 weeks and (**C**) 20 weeks; PLGCL+DTX/DCM patches: (**B**) 4 weeks and (**D**) 20 weeks.

**Figure 5:**
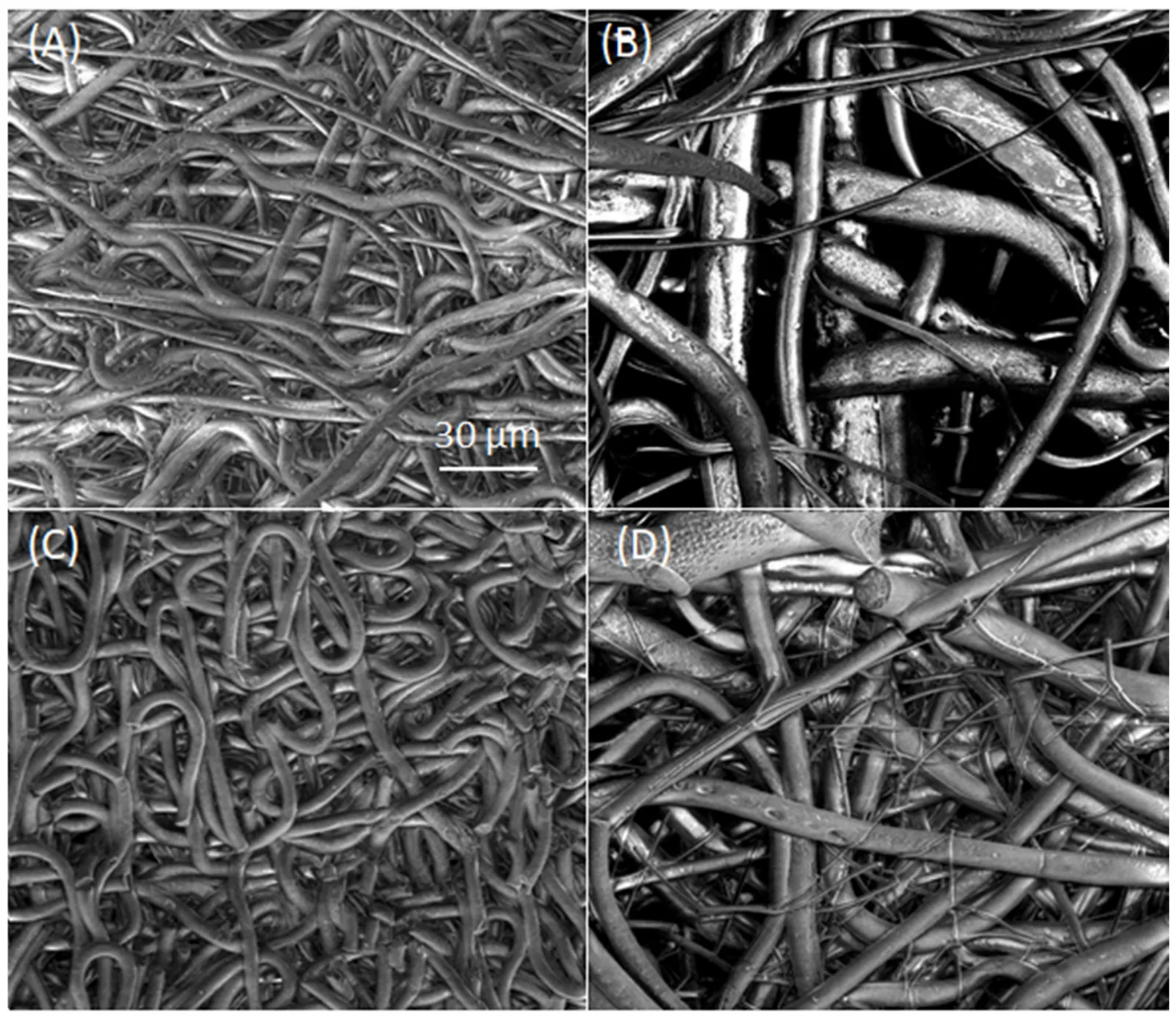
SEM images of drug-loaded patches during degradation (×2500, all scale bars are 30 µm): PLGCL+DTX/HFIP patches: (**A**) 4 weeks and (**C**) 20 weeks; PLGCL+DTX/DCM patches: (**B**) 4 weeks and (**D**) 20 weeks.

**Figure 6:**
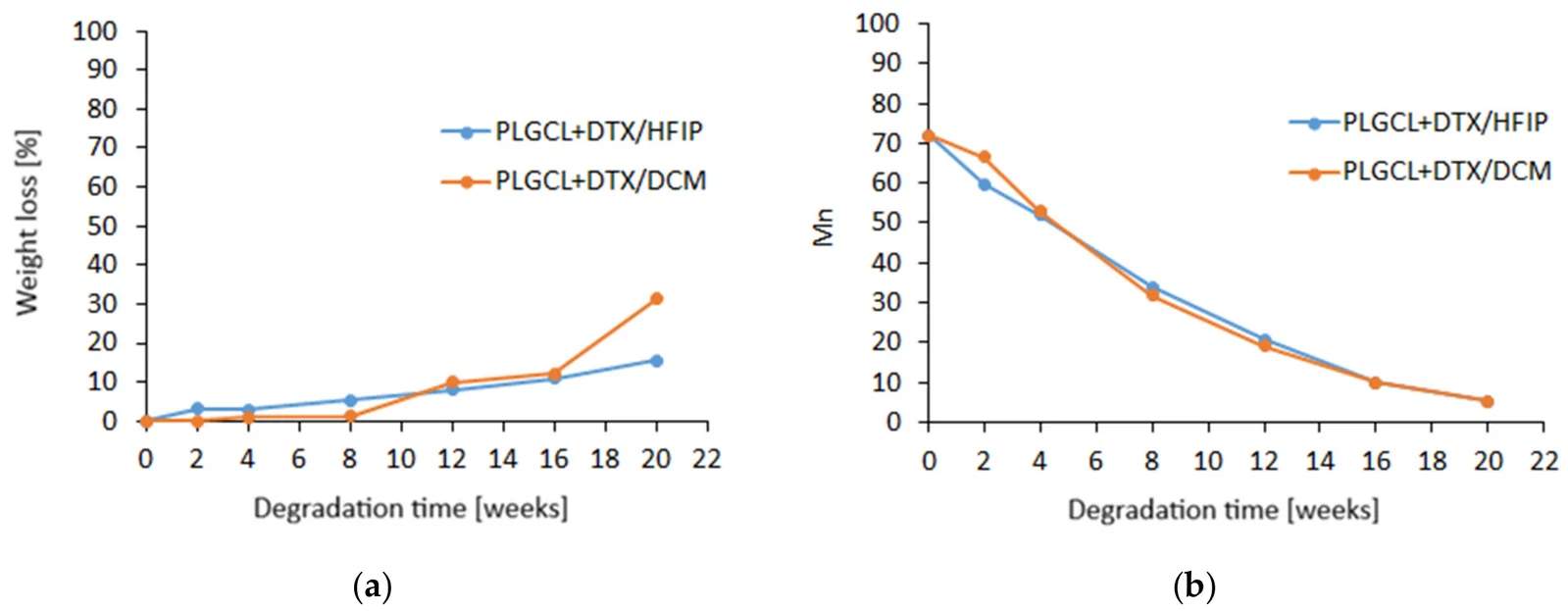
Weight loss (**a**), and changes in M_n_ (**b**) during degradation of drug-loaded patches (*n* = 3).

Changes in morphology were monitored by SEM (Figure 4 and Figure 5). Changes in the polymer were monitored by means of GPC and NMR. Moreover, weight loss was studied to assess the progress of the degradation. As shown in Figure 6a, in the different stages of degradation the weight loss was similar for all drug-loaded patches. However, at the end of the experiment, at the 20th week of degradation, the weight loss of PLGCL-DTX/DCM was much higher (31%) than in the case of PLGCL-DTX/HFIP (15.5%). The patches obtained with HFIP were quite stiff, with a rigid and compact structure, whereas patches obtained with DCM had a loose, delicate structure; thus, water could penetrate the inner parts more easily and they split into smaller parts.

**Figure 7:**
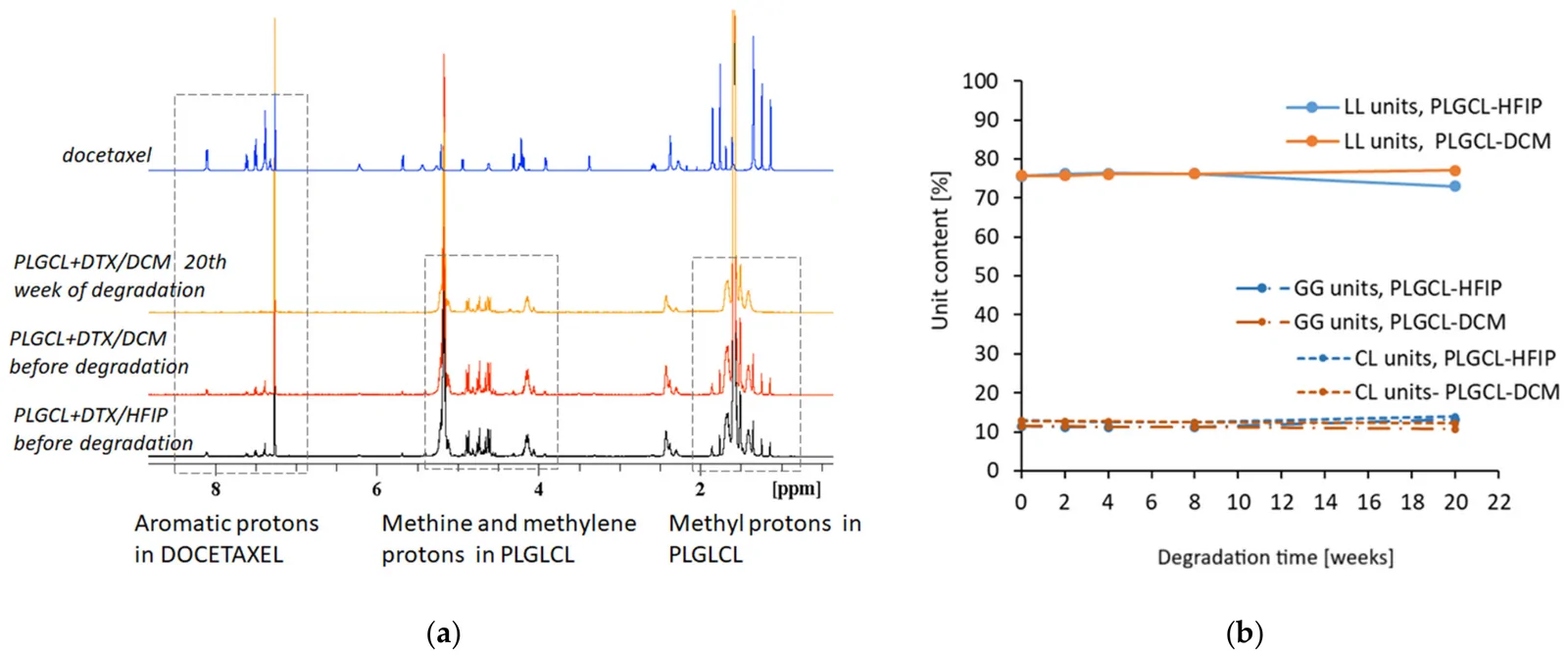
(**a**) ^1^H NMR spectra of PLGCL+DTX and pure DTX (CDCl_3_, 600 MHz); (**b**) changes in the percentage content of comonomer units (LL—lactidyl units, GG—glycolidyl units, CL—caproyl units) in poly(lactide-co-glycolide-co-ε-caprolactone) during 20 weeks of degradation.

Despite molar mass changes and weight loss, the changes in the unit content during degradation, evaluated based on NMR spectra, were negligible, indicating a stable course (Figure 7). Due to the random microstructure of the chain, developed during the synthesis reaction (held in bulk, according to the coordination/insertion mechanism) and due to using the Zr(acac)_4_ initiator, a regular and even course of degradation could be obtained. ^1^H NMR spectra detected at every stage of degradation of all the drug-loaded electrospun patches confirmed a gradual reduction in the drug content in the polymer chain. Figure 7 presents selected spectra. At the 20th week of degradation signals connected with docetaxel are no longer observed.

### 2.5. Anticancer Properties

#### 2.5.1. L929 Fibroblast Cytotoxicity Assay

**Figure 8:**
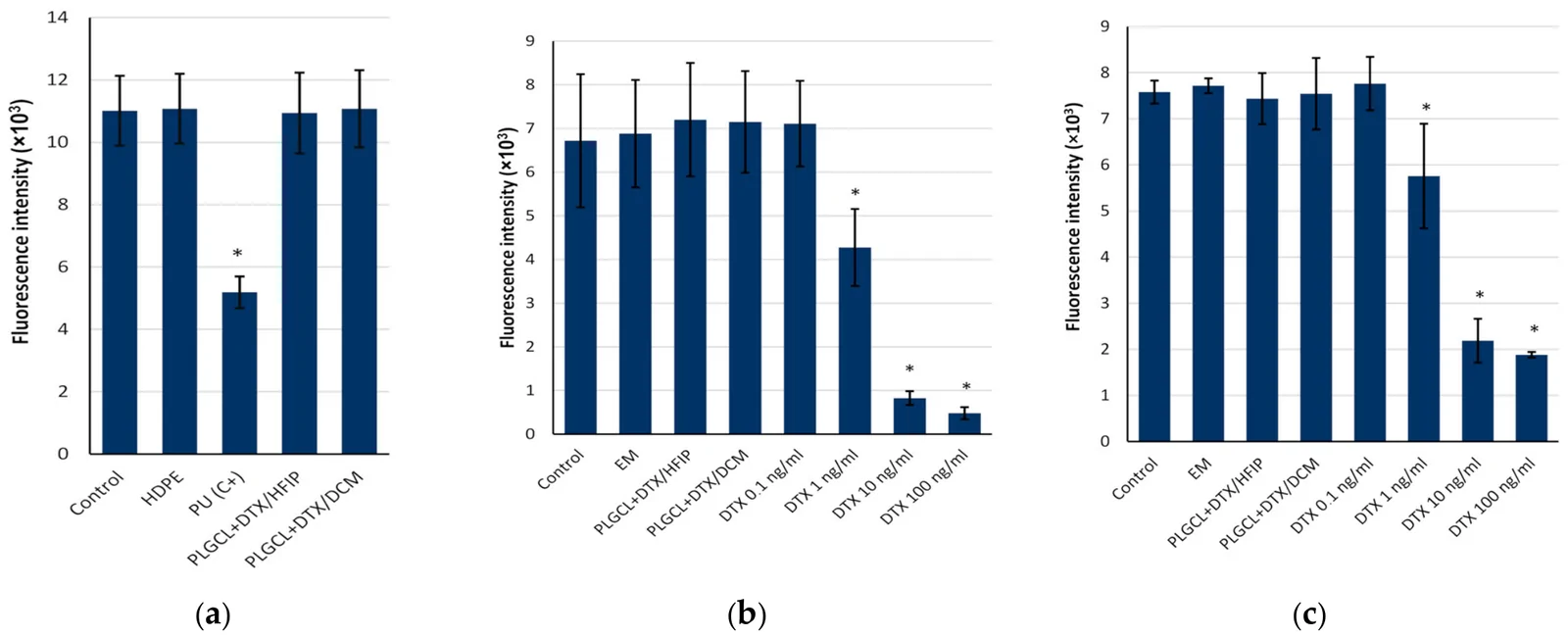
(**a**) Growth of L-929 cells in a direct contact with polymeric fibrous membranes; HDPE—high-density polyethylene, PU (C+)—polyurethane containing 0.1% ZDEC. Growth of (**b**) DU-145 and (**c**) PC-3 cells in the presence of extracts from drug-free polymeric patches and free docetaxel solutions; EM—extraction medium incubated without patch specimens; n = 3, ^∗^ *p* < 0.05 compared with control.

To consider a new biomaterial safe for patients, the possibility of any potential cytotoxic effects must be ruled out. In our study, the drug-loaded electrospun patches PLGCL+DTX/DCM and PLGCL+DTX/HFIP were placed in direct contact with L-929 cells, a standard experimental model for testing materials and medical devices. Due to the close proximity of the fibrous sample to the cell layer, any leachable cytotoxic substances diffusing out of the polymeric matrix can easily disturb cell viability and proliferation. Therefore, it is a sensitive and reliable technique enabling detection of even low levels of toxicity. As shown in Figure 8a, incubation of L-929 cells with the positive control (polyurethane containing 0.1% ZDEC) for 72 h resulted in an approximately 50% reduction in cell viability. Simultaneously, specimens of electrospun patches PLGCL+DTX/DCM and PLGCL+DTX/HFIP did not show any manifestations of cytotoxicity, similar to the negative control samples (HDPE).

#### 2.5.2. Antiproliferative Activity in Prostate Cancer Cells

Having confirmed the cytocompatibility of the polymeric carrier, we next analyzed the ability of drug-loaded electrospun patches to inhibit the growth of prostate cancer cells in vitro. Preparation of extracts from patches enables an evaluation of their cytotoxicity based on the degree of dilution at which the toxic effect disappears. As shown in Figure 8b,c, undiluted extracts from drug-free electrospun patches did not exert any cytotoxic effects on DU-145 and PC-3 cells. Simultaneously, solutions of free docetaxel caused a dose-dependent inhibition of cell proliferation, with IC50 values of 1.195 ng/mL for the DU-145 cell line and 1.501 ng/mL for the PC-3 cell line.

**Figure 9:**
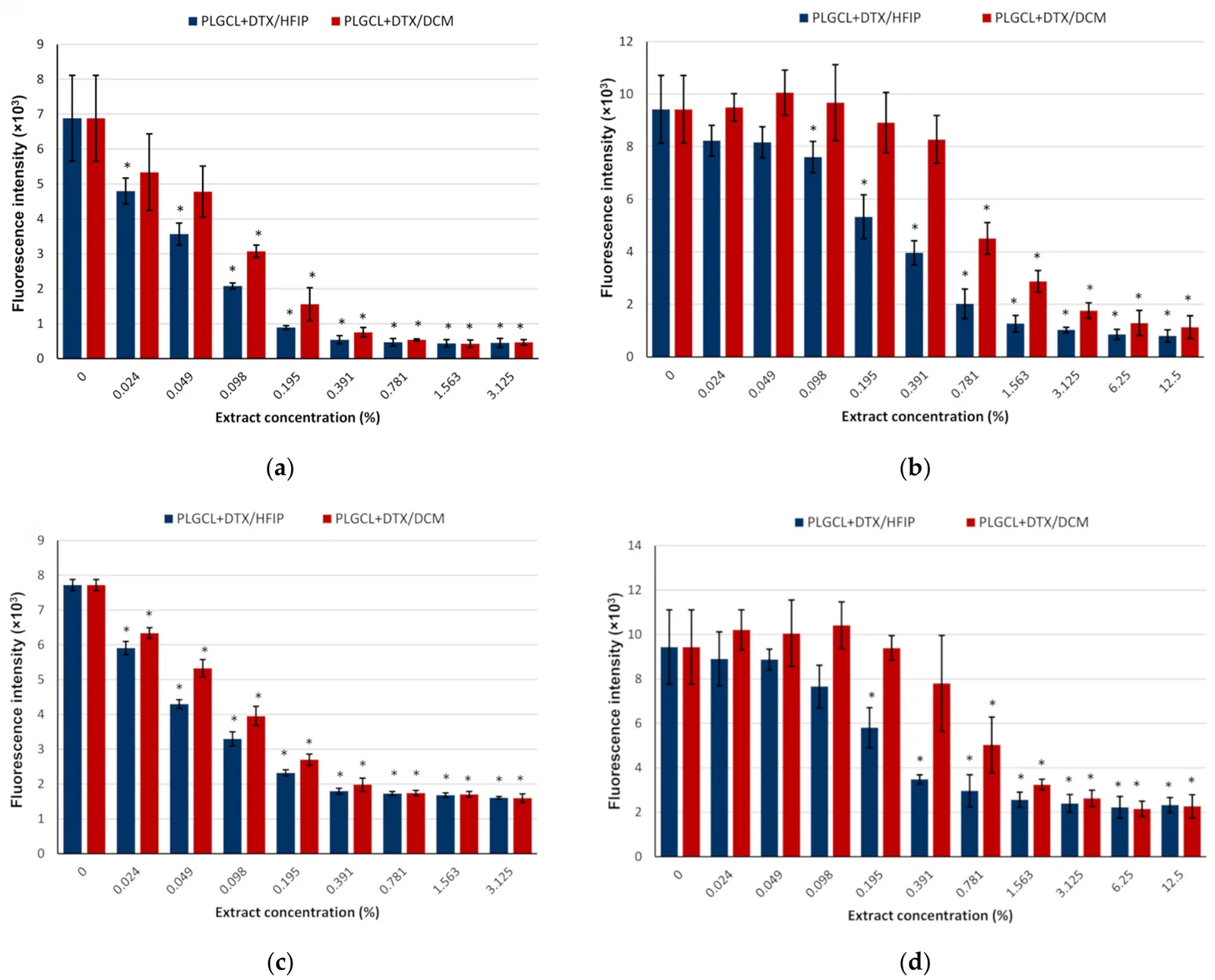
Antiproliferative activity of extracts from DTX-loaded PLGCL electrospun patches against DU-145 (**a**,**b**) and PC-3 (**c**,**d**) cells. Extracts were prepared from patches without preincubation (**a**,**c**) or after 14 days of preincubation in culture medium (**b**,**d**). Cells were exposed to serially diluted extracts for 72 h, and metabolic activity was assessed using the PrestoBlue assay. HFIP and DCM indicate the solvents used to prepare the membranes. Values are expressed as mean ± SD. * *p* < 0.05 compared with the corresponding control (0% extract).

Extracts prepared from drug-loaded electrsopun patches without preincubation exerted a strong cytotoxic effect on both prostate cancer cell lines (Figure 9a,c). Growth inhibition was noticeable even at the lowest applied extract concentration—0.024% for both tested electrospun patches. An increase in extract concentration augmented the cytotoxic effect up to 3.125% (dilution 32×). Higher concentrations of both extracts did not cause a stronger growth inhibition; therefore, they are not included in the charts. The efficacy of the drug-loaded electrospun patches obtained with the use of HFIP (PLGCL+DTX/HFIP) was slightly higher than patches fabricated using DCM (PLGCL+ DTX/DCM), as indicated by the IC50 values (0.056% and 0.08% for DU-145 cells respectively, together with 0.038% and 0.077% for PC-3 cells). These results demonstrate that during the initial 24 h extraction period, a sufficient amount of the anticancer drug was released from the tested patches to effectively inhibit cell proliferation.

To examine whether the antiproliferative activity persisted beyond the initial burst-release phase, extracts were also prepared from electrospun patches preincubated in culture medium for 14 days (Figure 9b,d). Both systems, PLGCL-DTX/HFIP and PLGCL+DTX/DCM, retained inhibitory activity against DU-145 and PC-3 cells, with the response shifted toward higher extract concentrations compared with extracts obtained without preincubation. For the PLGCL+DTX/HFIP patches, significant decreases in the PrestoBlue fluorescence signal were observed at extract concentrations of 0.098–12.5% in DU-145 cells and 0.195–12.5% in PC-3 cells. For the PLGCL+DTX/DCM patches, significant decreases occurred at concentrations of 0.781–12.5% in both cell lines. Higher concentrations of both extracts did not cause a stronger growth inhibition; therefore, they are not shown in the charts. Extracts obtained from preincubated patches prepared using HFIP were able to inhibit the growth of both cell lines at lower concentrations than those from patches prepared using DCM: the IC50 values were 0.259% and 0.643% for DU-145 cells, respectively, compared with 0.185% and 0.575% for PC-3 cells.

The persistence of inhibitory activity after 14 days of preincubation indicates that the patches retained the capacity to release biologically active docetaxel beyond the initial burst-release phase. The lower activity of the extracts obtained after preincubation is consistent with a somewhat reduced rate of drug release at this stage of the process (Figure 3). Together, these findings support the idea that drug-loaded electrospun patches obtained with the use of PLGCL terpolymer might be developed into a carrier for docetaxel characterized by efficient, long-term and stable antitumor action.

## 3. Discussion

Biodegradable platforms for anticancer drugs have been developed during recent years using different methods and materials. The most popular biodegradable polymers used for this purpose are poly(lactide-glycolide) and poly(lactide), which are connected not only due to their properties, but also due to commercial availability. A terpolymeric platform has rarely been used. However, incorporating three different kinds of structural units into the polymeric chain provides interesting materials with combined features. Poly(lactide-co-glycolide-co-ε-caprolactone) and docetaxel were used in the current study to prepare electrospun patches using two different solvents for electrospinning: hexafluoroisopropanol HFIP and dichloromethane DCM. The choice of solvent is important during electrospinning since it determines the structure and morphology of the fibrous drug delivery system, and has additional consequences for the whole system. However, it is worth mentioning that in order to obtain a good fiber-forming polymer solution for electrospinning, it is important to choose an appropriate concentration. This requires adjusting the process parameters; thus, they can differ for various solutions. In our study we focused on the properties of electrospun drug delivery systems prepared with two different solvents, with different polarity and volatility. It resulted in a different morphology of the fibers and, consequently, different characteristics of the whole electrospun drug delivery system, including the drug release profile. Other significant features, such as the course of degradation and anticancer properties, were also analyzed.

In the case of PLGCL+DTX/HFIP, fibers were more homogeneous than for PLGCL+DTX/DCM. During degradation, the morphology of the fibers changed and in the case of PLGCL+DTX/DCM, fibers swelled after incubation in PBS medium. Ding Q. et al. [24] prepared electrospun patches from poly-D,L-lactide (Mw =135,000) in DCM. The surfaces of the fibers were polyporate. Xing L. et al. [25] used two solvents, DCM and DMF (0.7/0.3), for electrospinning of poly(L-lactide) (Mw = 100,000) with 5% and 10% DTX. SEM micrographs revealed uniform nanofibers with smooth surfaces, without bead formation. In the case of the PLGCL+DTX/DCM presented in the current study, the porosity of the fibers was not noticed; however, in our previously published research we observed this. In order to characterize the interactions between the polymer platform and the encapsulated drug, which also plays an important role in delivering the drug, FTIR analysis for the docetaxel delivery systems was conducted in our study. The analysis indicated the formation of hydrogen bonds between the carbonyl ester groups of the terpolymer and the urethane groups of the docetaxel. The observed effect was higher for the system obtained with DCM (PLGCL+DTX/DCM). HFIP is known for its hydrogen bond donating ability [26]; however, different interactions occur during the electrospinning process, resulting in the final structure of the nonwovens. They are connected with the affinity of the solvent not only toward polymer but also toward drug molecules.

The courses of degradation of both electrospun systems, PLGCL+DTX/HFIP and PLGCL+DTX/DCM, were similar: the weight loss and molar mass changes did not differ, except for the last time point, on the 20th week. However, changes in the drug release profile were observed. In both systems, a burst effect occured. The burst release of the drug is usually caused by its accumulation on the surface of the fibers [6], which might be the effect of solvent evaporation. In this study, the release of the drug from PLGCL+DTX/HFIP was more rapid, especially in the first two weeks of incubation. A possible explanation might be the migration of drug molecules; when the solvent, whose affinity toward the drug is high, evaporates during the electrospinning process, the drug molecules migrate with the solvent to the surface and accumulate on it, resulting in burst release. This might be the reason for the higher burst effect observed in the case of PLGCL+DTX/HFIP. On the other hand, the lower burst effect for DCM observed in this study was supported by the FTIR study, where hydrogen bond formation between polymer and drug was confirmed (more advanced than for HFIP). For both systems presented in this study, the entire amount of docetaxel was released within 20 weeks. The HPLC analysis was confirmed by NMR spectra, where signals connected with docetaxel compounds (especially observed in the aromatic region of the protons) diminished in the 20th week of incubation. Burst effect was consciously used to achieve a better therapeutic efficiency in a study by Liang et al. [27]. They developed a biphasic drug-release postoperative in situ gel system, which integrated rapid and sustained release to inhibit the recurrence of glioma. This strategy inhibited the glioma stem cells, and regulated the postoperative recurrence microenvironment. The developed gels exhibited an initial burst release of about 40–50% within the first 120 h— it created a highly cytotoxic zone around the resection cavity, attributed to the rapid diffusion of free drugs in the gel, followed by controlled released from PLGA nanoparticles.

The cytocompatibility of the obtained electrospun drug-loaded patches was also analyzed. For both PLGCL+DTX/HFIP and PLGCL+DTX/DCM, any manifestations of cytotoxicity were not observed, similar to the negative control samples. Finally, the antiproliferative activity of the drug-loaded electrospun patches was evaluated on prostate cancer cells: DU-145 and PC-3 cell lines. They exerted a strong cytotoxic effect on both prostate cancer cell lines. Growth inhibition was noticeable even at the lowest applied extract concentration. Moreover, in a series of additional experiments using extracts from electrospun drug-loaded patches, preincubated in culture medium for 14 days, we showed that the patches retained the capacity to release biologically active docetaxel with antiproliferative effects beyond the initial burst-release phase. Therefore, it can be expected that electrospun drug delivery systems in the form of flexible patches obtained with the use of PLGCL terpolymer can be developed into a carrier for docetaxel characterized by efficient and stable antitumor action. Thus, the reported results provide a rationale for progressing to more advanced stages of preclinical drug evaluation, including the assessment of therapeutic efficacy in animal models of experimentally induced tumors.

## 4. Materials and Methods

### 4.1. Synthesis of Poly(L,L-lactide-co-glycolide-co-ε-caprolactone)

Poly(lactide-co-glycolide-co-ε-caprolactone) (75/10/15) was synthesized and applied as an electrospun drug delivery system. The monomers L-lactide and glycolide (both Foryou Medical Devices, Huizhou, China) were purified by recrystallization from dried ethyl acetate and subsequently dried in a vacuum oven at room temperature. ε-Caprolactone (Acros Organics, Geel, Belgium) was purified by vacuum distillation over freshly powdered calcium hydride prior to use. The terpolymer was synthesized via ring-opening polymerization (ROP) of L-lactide (90 g; 0.625 mol) glycolide (9.63 g; 0.083 mol) and ε-caprolactone (14.25 g; 0.125 mol) using zirconium(IV) acetylacetonate (Zr(acac)_4_; Merck, Darmstadt, Germany) (0.339 g; 0.694 mmol) as the initiator. The detailed procedure for preparing poly(lactide-co-glycolide-co-ε-caprolactone) was described by Dobrzynski [25]. The molar ratio of Zr(acac)_4_ to total monomers was 1:1200. Polymerization was carried out in bulk at 120 °C for 96 h under an argon atmosphere. The resulting terpolymer was purified by dissolving in chloroform, followed by precipitation in methanol (both Merck, Darmstadt, Germany), and then vacuum-dried to constant weight.

### 4.2. Preparation of Electrospun Drug Delivery Systems

The nonwoven drug delivery systems were obtained with a TL-Pro-BM electrospinning unit (Tong Li Tech, Shenzhen, China) which was equipped with two high-voltage power supplies. One of them was applied to the spinneret (generates positive electrical potential), and the second was applied to the fiber collector (generates negative potential). A mandrel of 27 mm diameter was used as a rotating fiber collector. Poly(lactide-co-glycolide-co-ε-caprolactone) and docetaxel (LC Laboratories, Woburn, MA, USA) were dissolved in hexafluoroisopropanol HFIP (1st group of drug-loaded materials) and dichloromethane DCM (2nd group of drug-loaded materials). HFIP was purchased from Fluorochem, Hadfield, UK, and DCM from Avantor, Gliwice, Poland. The concentration of the HFIP solution was 8% and 12% of the DCM solution. The drug/polymer ratio was 1:19. Polymer solutions were dosed to the spinning nozzle using a PHD Ultra 4400 syringe pump (Harvard Apparatus, Holliston, MA, USA). Electrospun drug delivery systems were produced according to process parameters, presented in Table 2. The electrospinning chamber allowed keeping the temperature in the range of 17–18 °C and relative humidity at 21%. DCM and HFIP slowly evaporated at room temperature. After this, drug-loaded mats were dried under vacuum (80 Mbar for 14 days). Patches with 1 cm diameter were cut out.

**Table 2:** Parameters of the electrospinning process.

Process Parameter	PLGCL+DTX/HFIP	PLGCL+DTX/DCM
Positive voltage [kV]	10.5	17.5
Negative voltage [kV]	−7.5	−12.0
Distance between the spinneret and collector [cm]	23.0	28.0
Feed rate [ml/h]	3.0	2.0
Needle gauge	G20	G20

#### Analysis of Physicochemical Properties of Electrospun Patches

The properties of the drug-loaded electrospun patches were characterized using the following techniques:

- ^1^H Nuclear Magnetic Resonance Spectroscopy (600 MHz Bruker Avance II Ultrashield Plus, Bruker, Billerica, MA, USA) was used to analyze comonomer unit content in the polymer chain of the drug-loaded patches. Spectra were recorded with 128 scans, 11 µs pulse width, and 2.65 s acquisition time. CDCl_3_ was used as a deuterated solvent with TMS internal standard.
- Gel Permeation Chromatography (Physics SP 8800 Milpitas, Palo Alto, CA, USA, chromatograph equipped with Styragel columns and Shodex SE 61 detector) was used to analyze the molar mass of the polymer of the drug-loaded patches. The following conditions were used: eluent: CHCl_3_, flow rate: 1 mL/min, polystyrene standards.
- Fourier Transform Infrared Spectroscopy (Jasco FT/IR-6000 spectrometer, Jasco, Tokyo, Japan) was used to analyze polymer/drug interactions. An ATR attachment for the foils was used and a KBr pellet for the DTX sample.
- Scanning Electron Microscopy (SEM, Quanta 250 FEG, FEI Company, Hillsboro, OR, USA) was used to analyze the morphology of the electrospun patches. The following conditions were used: low vacuum conditions (80 Pa), an acceleration voltage of 5 kV from secondary electrons collected by a Large Field Detector.

### 4.3. Degradation Study

The degradation study was conducted for 140 days at 37 °C. Drug-loaded patches with a diameter of 1 cm were incubated in 5 mL of PBS with Tween 80 (0.5%; *v*/*v*), pH 7.4, under constant agitation at 240 rpm. To maintain sink condition, PBS buffer was replaced every 7 days. On the basis of this study, the percentage of weight loss (W_L_ %) was calculated. The following equation was used for the calculation: W_L_(%) = [(W_0_ − W_dry_)/W_0_] ∗ 100 (W_0_: initial weight, and W_dry_: residual weight). The degradation process was also characterized using Gel Permeation Chromatography and ^1^H Nuclear Magnetic Resonance Spectroscopy.

### 4.4. Drug Release Study

Drug release was investigated for 140 days, at 37 °C. Drug-loaded patches with a diameter of 1 cm were incubated in 5 mL of PBS with pH 7.4 with Tween 80 (0.5%; *v*/*v*) under constant agitation at 240 rpm. To maintain sink condition PBS buffer was replaced every 7 days (and additionally on the 3rd day). A high-performance liquid chromatograph (VWR-Hitachi/Chromaster^®,^ Merck, Germany) equipped with a LiChrospher^®^ RP-18 column (250 mm × 4 mm, 5 μm) and LiChrospher^®^ RP-18 guard column (4 mm × 4 mm, 5 μm) was used for evaluating the amount of released drug (docetaxel was detected at a wavelength of 227 nm) on the basis of the extraction method described in detail in Section 2.3. The mobile phase consisted of acetonitrile and water (60:40, *v*/*v*). The flow rate was 1 mL/min.

### 4.5. Anticancer Activity

#### 4.5.1. Cell Lines and Culture Maintenance

All cell lines used in this study were obtained from the American Type Culture Collection (ATCC, Manassas, VA, USA). Non-cancerous cells (the L-929 mouse subcutaneous fibroblast cell line, ATCC CCL-1^TM^) were cultured in ATCC-formulated Eagle’s Minimum Essential Medium (MEM). Human prostate cancer PC-3 cells (ATCC CRL-1435^TM^) were cultured in F-12K medium (Merck, Poznan, Poland) and the DU-145 prostate cancer cell line (ATCC HTB-81^TM^) was maintained in Eagle’s MEM (Merck, Poznan, Poland). All culture media were supplemented with 10% fetal bovine serum (FBS, Thermo Fisher Scientific, Waltham, MA, USA), 100 U/mL penicillin, 100 µg/mL streptomycin and 10 mM HEPES (Merck, Poznan, Poland). The cells were cultured at 37 °C in a humidified atmosphere containing 5% CO_2_, supplied by the MCO-19AICUV-PE CO_2_ incubator (Panasonic Healthcare, Osaka, Japan). Cells were passaged using 0.25% trypsin/0.02% EDTA solution (Merck, Poznan, Poland).

#### 4.5.2. Assessment of Cytocompatibility Using the Direct Contact Test

The release of substances from the drug-loaded patches is controlled by a diffusion process and occurs at the surface. To prepare samples with a specified, constant surface area the nonwoven mats were cut into small disks (5 mm in diameter) with the aid of a cork borer. The cytocompatibility of the drug-free electrospun mats was studied using a method adapted from the IS0 10993-5 standard [28]. High-density polyethylene was used as a non-toxic reference material (negative control) and polyurethane containing 0.1% zinc diethyldithiocarbamate (ZDEC) was applied as a cytotoxic reference material—positive control (both purchased from the Hatano Research Institute, FDSC, Kanagawa, Japan). The assay was based on determining the viability of L-929 cells following direct contact with electrospun mats. Fibroblasts were seeded into 24-well plates (Corning, Corning, NY, USA) at an initial density of 5 × 10^4^ cells per well in 1 mL of culture medium and incubated for 24 h to enable cell attachment. Then, the culture medium was replaced with a fresh one and circular material specimens were placed directly on the cell layer, in the center of wells. Fibroblasts were cultured in the presence of electrospun mats for 72 h and their viability was determined by means of the PrestoBlue reagent (Thermo Fisher Scientific, Waltham, MA, USA), whose main ingredient is resazurin. The reduction of this compound by living cells, to a pink product—resorufin—was assessed fluorometrically using the Infinite^®^ 200 PRO multimode plate reader (Tecan, Männedorf, Switzerland), with an excitation wavelength of 560 nm and an emission wavelength of 590 nm.

#### 4.5.3. Cytotoxicity Evaluation Against Prostate Cancer Cells

To determine in vitro the cytotoxic activity of docetaxel incorporated into polymeric electrospun mats, circular specimens with a diameter of 5 mm were placed in the complete culture medium and extracted for 24 h at 37 °C, with constant agitation (240 rpm). The volume of the incubation medium was adjusted to the surface area of the electrospun mats to obtain a surface area-to-volume ratio of 10 mm^2^/mL. Prepared extracts were serially diluted with fresh medium in a 1:1 proportion, up to a dilution of 1:4096. Concomitantly, extracts from drug-free mats and solutions of free docetaxel in culture medium (acting as a positive control) were fabricated. To exclude any possible negative effect of the extraction process on the properties of the culture medium, an additional control group was prepared, where the medium was incubated without electrospun mats. In a separate experiment, DTX-loaded electrospun mats were preincubated in culture medium for 14 days before extraction. This step was intended to remove the contribution of the initial burst release and assess the antiproliferative activity of drug released during the subsequent stationary phase. After preincubation, the medium was discarded and the electrospun mats were transferred to fresh complete culture medium for a further 24 h extraction under the conditions described above. These extracts were then serially diluted.

Prostate cancer cells were plated into black 96-well microplates (Greiner Bio-One, Kremsmünster, Austria) at a density of 5 × 10^3^ cells/well (in 200 µL of medium) and cultured for 24 h. Then, the culture medium was removed and replaced with the extracts or docetaxel solutions, followed by a 72 h incubation. Cell growth and viability were assessed with the use of the PrestoBlue reagent as described above. Statistical analysis was performed with the use of Statistica 13.1 software (StatSoft, Tulsa, OK, USA). The data are presented as the mean ± SD of results from three independent experiments (each including 4 technical replicates). They were statistically analyzed using randomized block ANOVA (RB-ANOVA) [29,30] followed by Tukey’s post hoc test. IC50 values were calculated with the use of GraphPad Prism 10 software with non-linear regression.

## 5. Conclusions

Poly(lactide-co-glycolide-co-ε-caprolactone) and docetaxel were used in the current study to prepare electrospun patches using two different solvents for electrospinning: hexsafluoroisopropanol HFIP and dichloromethane DCM. It resulted in different morphology of the fibers and different characteristics of the whole electrospun drug delivery system, including the drug release profile. For both systems, the entire amount of docetaxel was released within 20 weeks. Interactions between the polymer platform and the encapsulated drug were confirmed: hydrogen bonds between the carbonyl ester groups of the terpolymer and the urethane groups of the docetaxel were formed. The courses of degradation of both electrospun systems were similar: the weight loss and molar mass changes did not differ, except at the last time point. During degradation, the morphology of the fibers changed and in the case of PLGCL+DTX/DCM the fibers gradually swelled following incubation in PBS medium. Drug-loaded electrospun systems exerted a strong cytotoxic effect on the DU-145 and PC-3 prostate cancer cell lines. It can be concluded that the results of this study support further investigation of electrospun patches based on PLGCL terpolymer as potential carriers for local docetaxel delivery in prostate cancer.

## Figures and Tables

**Table 1:** The comparison of different procedures used for drug loading evaluation.

Procedure	Efficiency [%]							
	PLGCL+DTX/HFIP				PLGCL+DTX/DCM			
	1	2	3	Average	1	2	3	Average
(1) 1 mL DCM + 9 mL EtOH	87.6	88.2	88.2	88.0	87.4	86.8	86.2	86.8
(2) 10 mL ACN	95.6	96.4	95.6	95.9	93.0	93.4	94.2	93.5
(3) 5 mL ACN	94.4	94.4	95.2	94.7	93.6	93.0	92.2	92.9

## Data Availability

The dataset is available on request from the authors.
